# Results of Tibial sesamoid planing

**DOI:** 10.1186/1757-1146-3-S1-O14

**Published:** 2010-12-20

**Authors:** Ewan Kannegieter

**Affiliations:** 1 Derbyshire County PCT, Derbyshire, UK

## Introduction

Tibial sesamoid planing is often used for the treatment of tibial sesamoiditis and intractable plantar keratosis. This presentation will review the literature and present the details of a retrospective study of patients who have undergone tibial sesamoid planing.

## Methods

Departmental database revealed 46 patients who have undergone tibial sesamoid planing between May 1996 to Sept 2009. These patients were invited for an audit appointment, to assess outcomes both subjective and objective.

## Results

Study is ongoing.

## Discussion

There is a lack of evidence pertaining to this treatment in the literature.

Clinical and radiological data, pre and post-operative AOFAS scores, along with patient centred outcomes will be reported.

